# Validation of nonrigid registration in pretreatment and follow‐up PET/CT scans for quantification of tumor residue in lung cancer patients

**DOI:** 10.1120/jacmp.v15i4.4847

**Published:** 2014-07-08

**Authors:** Jolanda Spijkerman, Davide Fontanarosa, Marco Das, Wouter van Elmpt

**Affiliations:** ^1^ Department of Radiation Oncology (MAASTRO) GROW – School for Oncology and Developmental Biology, Maastricht University Medical Centre Maastricht The Netherlands; ^2^ Oncology Solutions Philips Research Eindhoven The Netherlands; ^3^ Department of Radiology GROW – School for Oncology and Developmental Biology, Maastricht University Medical Centre Maastricht The Netherlands

**Keywords:** PET, deformable algorithm, pattern of relapse

## Abstract

Nonrigid registrations of pre‐ and postradiotherapy (RT) PET/CT scans of NSCLC patients were performed with different algorithms and validated tracking internal landmarks. Dice overlap ratios (DR) of high FDG‐uptake areas in registered PET/CT scans were then calculated to study patterns of relapse. For 22 patients, pre‐ and post‐RT PET/CT scans were registered first rigidly and then nonrigidly. For three patients, two types (based on Demons or Morphons) of nonrigid registration algorithms each with four different parameter settings were applied and assessed using landmark validation. The two best performing methods were tested on all patients, who were then classified into three groups: large (Group 1), minor (Group 2) or insufficient improvement (Group 3) of registration accuracy. For Group 1 and 2, DRs between high FDG‐uptake areas in pre‐ and post‐RT PET scans were determined. Distances between corresponding landmarks on deformed pre‐RT and post‐RT scans decreased for all registration methods. Differences between Demons and Morphons methods were smaller than 1 mm. For Group 1, landmark distance decreased from 9.5 ± 2.1 mm to 3.8 ± 1.2 mm (mean ± 1 SD, *p* < 0.001), and for Group 3 from 13.6 ± 3.2 mm to 8.0 ± 2.2 mm (p=0.02). No significant change was observed for Group 2 where distances decreased from 5.6 ± 1.3 mm to 4.5 ± 1.1 mm (p=0.02). DRs of high FDG‐uptake areas improved significantly after nonrigid registration for most patients in Group 1. Landmark validation of nonrigid registration methods for follow‐up CT imaging in NSCLC is necessary. Nonrigid registration significantly improves matching between pre‐ and post‐RT CT scans for a subset of patients, although not in all patients. Hence, the quality of the registration needs to be assessed for each patient individually. Successful nonrigid registration increased the overlap between pre‐ and post‐RT high FDG‐uptake regions.

PACS number: 87.57.Q‐, 87.57.C‐, 87.57.N‐, 87.57.‐s, 87.55.‐x, 87.55.D‐, 87.55.dh, 87.57.uk, 87.57.nj

## I. INTRODUCTION

Although prognosis of lung cancer patients has improved over the last decade, it remains one of the most lethal solid tumors. The five‐year survival is approximately 20% in patients with locally advanced non‐small cell lung cancer (NSCLC) treated with radiotherapy (RT), but, in the majority of patients, local relapses are observed after treatment.^1^, ^2^, ^3^ In order to improve treatment in future and design strategies to prevent local relapses, follow‐up scans of lung cancer patients are used to accurately quantify tumor treatment response patterns.

To find patterns of relapse, rigid registration between pretreatment (pre‐RT) and follow‐up posttreatment (post‐RT) positron emission tomography (PET)/computerized tomography (CT) scans was used mainly.^4^, ^5^, ^6^ Previous work that only used rigid registrations showed that the high FDG‐uptake areas prior to treatment largely corresponded to tumor residue areas.^(7)^ However, patients with significant anatomy changes during RT (e.g., large tumor shrinkage or deformation, weight loss or change of body position during scanning) had to be excluded. In this work, we propose a solution that uses deformable (nonrigid) registration to correct for such anatomical changes and allows investigation of the treatment response on a voxel‐to‐voxel level. Deformable registration was previously used to register CT scans in different respiratory phases for tumor tracking,^8^, ^9^ or for dose accumulation.^10^, ^11^ However, to our knowledge this is the first study that investigates deformable registration of follow‐up CT scans of lung cancer patients taken more than four months after pre‐RT scans.

With deformable registration, it is paramount to validate the deformation fields. Validation can be performed by defining landmarks on visible features inside the thorax (e.g., bifurcating vessels, calcifications, distinct anatomical shapes) in order to quantify the accuracy of the deformation fields.^12^, ^13^, ^14^

The verified registration fields can then be used to investigate the relation between high [18F]‐fluorodeoxyglucose (FDG)‐uptake areas in the tumor before the treatment and tumor residue three months after treatment. FDG‐uptake could serve as an indication of treatment‐resistant regions inside the tumor.^(6)^ In this research, the verified deformable registration fields calculated on the CT scans were applied to the PET scans. Since deformable registration could compensate for the anatomical changes that occur in the months between the scans, we hypothesized that the overlap between high FDG‐uptake areas is increased compared to rigid registration.

To test this hypothesis, we have two aims for this study: first, to validate deformable image registration between pre‐ and post‐RT PET/CT image datasets of NSCLC patients;. second, if accurate deformable registration is possible, to test the hypothesis that deformable registration techniques yield a better match than rigid transformation. For this, we evaluated Dice overlap ratios (DR) of tumor residue in the post‐RT scans with high FDG‐uptake areas in the pre‐RT PET scan. This research is a prerequisite for calculating voxel‐by‐voxel correspondence used in evaluating dose‐painting strategies.

## II. MATERIALS AND METHODS

### A. Patient data

Twenty‐two patients (six female and 16 male) were included, with inoperable NSCLC, UICC stage I‐III, treated with radical RT alone or with sequential chemo‐RT, according to the institutional protocol.^(6)^ The datasets are described comprehensively elsewhere.^(6)^ No treatment was given to any of the patients between the end of RT and the post‐RT scan. A PET/CT scanner (Siemens Biograph 40, Knoxville, TN) was used to image the patients, in treatment position. The CT part of the scan was acquired using 4D CT, the PET was acquired using 3D PET. The scans used in the analysis were in the midventilation phase of the 4D CT, the same phase was also used for attenuation correction of the PET scan.^(15)^ Two PET/CT scans were acquired: one before RT treatment and one approximately three months after treatment. The CT scans used in this study were noncontrast‐enhanced. Resolution of CT scans ranged from $0.7\times 0.7\times 3\;{\text{mm}}^{3}$ to $1\times 1\times 5\;{\text{mm}}^{3}$. For all PET scans the voxel size was $5.3\times 5.3\times 5\;{\text{mm}}^{3}$.

### B. Deformable registration and extensive validation

If a large initial mismatch between pre‐ and post‐RT CT scans was present, the scans were initially matched using automatic rigid registration. This automatic registration mainly matches the patient's contour in both scans. However, due to weight loss, for example, the size and shape may change. Therefore, for all patients, a manual rigid registration was applied to optimize the match between lung contours. A volume of interest (VOI) was created as the smallest box encompassing the PTV in the pretreatment scan with a further expansion of 20 mm. Since the computation time of nonrigid registration fields strongly depends on the image size, deformable registration was only calculated inside this VOI. In order to get a reliable registration field, the cropped pre‐ and post‐RT scans should both contain the same anatomical structures. Twenty millimeters is the minimal suitable margin for this purpose, according to our experience. Both the rigid and deformable registrations were performed using in‐house‐developed software.^(16)^

**Figure 1:**
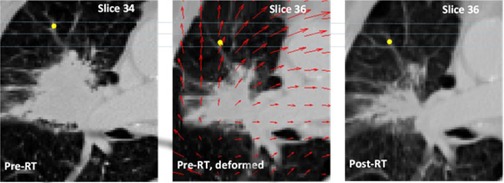
Representative image for a patient in Group 1 showing a landmark in the pre‐RT scan (left image) and post‐RT CT scan (right image, after rigid registration), the deformed pre‐RT CT scan is shown in the middle. Notice that there is also a difference in craniocaudal distance indicated by the slice numbers. Originally the landmarks in pre‐ and post‐RT scan were two slices apart, but after deformation they are located on the same slice. The deformation field is indicated by red arrows in the deformed scan. Note that the arrows point in the opposite direction of the displacement vectors. The landmark distance was reduced from 14 mm to 3 mm after deformable registration.

For three randomly selected patients, eight deformable registration fields were calculated to deform the pre‐RT CT scan to match the post‐RT CT scan. These fields were based on the Demons or Morphons methods, with 10 or 20 iterations per scale (eight resolution scales, each scale is an increase of factor 2, up to the full resolution^(10)^). A weighted sum accumulation (e.g., every iteration updates the previous calculation) or a diffeomorphic (e.g., invertible and smooth) accumulation of the deformation field was performed with a regularization filter (e.g., Gaussian smoothing of the calculated deformation vectors) of 1.5 times the voxel size. The final calculation scale was the full CT resolution. Demons is a fast algorithm based on image intensity differences. Morphons is based on intensity phase differences (e.g., local intensity gradients in the image). Details on the exact parameter setting for these two types of registration methods and four different choices of parameter settings are shown in Table 1. More details of the methods are described in the literature.^17^, ^18^ The landmarks in the deformed CT datasets of these three patients were extensively investigated to determine which deformation algorithms performed best. Together with an experienced radiologist, we annotated both the pre‐RT and post‐RT CT scan using approximately 30 landmarks/fiducials per patient. This included approximately ten landmarks inside or directly surrounding the primary lung tumor. These landmarks were selected based on visible features inside the thorax (e.g., bifurcating vessels, calcifications and distinct anatomical shapes). In Fig. 1, one landmark is reported as an example. As a measure of accuracy, the landmarks were tracked in the deformed scans, and absolute differences between deformed and nondeformed annotated landmark positions were calculated and compared for the various algorithms. It is important to notice that this validation method determines the accuracy of the registration field for a particular patient, not the accuracy of the deformation algorithms, as such.

Based on accuracy and computation times obtained with these three patients, we selected two parameter sets for deformable registration field calculation for the remaining 19 patients, one parameter set optimized for the Demons algorithm and one for the Morphons. For both algorithms, ten iterations per resolution scale and weighted sum accumulation of the update field were used. For these additional 19 patients, the accuracy of the two deformable registration fields was checked by annotating and tracking ten landmarks per patient.

Validation of rigid and deformable registration was quantified using the average absolute distance (3D vector) between the landmarks in the (deformed) pre‐ and post‐RT scans. The patients were then classified into three groups based on the following criteria:

- Group 1: Large improvement of the match between the pre‐ and post‐RT scans after deformable registration, compared to rigid registration; mean distance after deformable registration is less than half of the distance after rigid registration; mean distance after deformable registration is smaller than 6 mm.
- Group 2: No or minor improvement of the match between the pre‐ and post RT scans after deformable registration; mean distance after deformable registration is more than half of the distance after rigid registration; mean distance after rigid registration is smaller than 7.5 mm; mean distance after deformable registration is smaller than 6 mm.
- Group 3: Inadequate match after rigid and deformable registration; mean distance after rigid registration is larger than 7.5 mm; mean distance after deformable registration is larger than 6 mm.

The minimal distance of 6 mm is comparable to the PET resolution ($5.3\times 5.3\times 5\;{\text{mm}}^{3}$), whereas the maximal distance was chosen as approximately 1.5 times the PET resolution as an upper limit of the necessary registration accuracy.

**Table 1:** Absolute mean landmark distances and landmark distance range (smallest to largest distance), for the three patients after rigid and deformable registration. The applied algorithm features are described as Algorithm (rigid, based on Demons or based on Morphons), weighted sum/diffeomorphic field accumulation (W/D), and number of iterations

	*Algorithm*	*W/D*	*Iterations*	Mean ± SD *Entire VOI [mm] (range)*	Mean ± SD *Tumour [mm] (range)*	*Calculation Time*
Patient 1 31 Landmarks, 10 near tumour	Rigid			5.7±2.1 (1.0−10.2)	5.6±2.1 (2.8−8.5)	
	Demons	W	10	4.2±2.1 (1.0−7.6)	4.3±2.1 (1.0−6.6)	10min
	Demons	D	10	3.6±2.5 (1.0−10.9)	3.2±2.2 (1.0−7.6)	43min
	Demons	W	20	3.8±2.2 (1.0−10.2)	3.6±2.1 (1.0−6.6)	1h 12 min
	Demons	D	20	3.8±2.3 (1.0−10.1)	3.4±2.4 (1.0−7.6)	2h 34min
	Morphons	W	10	3.3±2.7 (0−10.6)	3.2±1.9 (1.0−5.4)	18min
	Morphons	D	10	3.6±3.3 (0−15.3)	3.0±1.9 (1.0−5.4)	1h 3min
	Morphons	W	20	3.5±2.7 (0−10.6)	3.1±1.9 (1.0−5.4)	2h 53min
	Morphons	D	20	3.2±2.5 (0−10.2)	2.8±1.8 (1.0−5.2)	4h 17 min
Patient 2 30 landmarks, 12 near tumour	Rigid			8.7±4.2 (3.0−17.2)	9.7±3.9 (3.0−16.1)	
	Demons	W	10	7.2±3.1 (1.0−11.5)	6.8±3.2 (3.0−11.5)	11min
	Demons	D	10	6.5±3.4 (1.0−12.6)	6.0±4.0 (1.0−12.6)	60min
	Demons	W	20	7.2±3.2 (0−11.5)	6.9±3.1 (3.0−11.5)	1h 20min
	Demons	D	20	6.3±3.6 (0−12.6)	5.9±4.2 (1.0−12.6)	3h 9min
	Morphons	W	10	4.4±2.6 (0−9.5)	3.0±2.2 (0−7.9)	27min
	Morphons	D	10	5.6±3.2 (0−10.7)	4.9±3.4 (0−10.7)	51min
	Morphons	W	20	4.5±2.4 (0−8.6)	3.1±2.0 (0−7.4)	1h 50min
	Morphons	D	20	3.7±1.9 (0−6.9)	2.1±1.9 (0−5.5)	3h 53min
Patient 3 32 landmarks, 11 near tumour	Rigid			8.9±4.2 (2.2−17.1)	10.2±4.5 (2.2−17.1)	
	Demons	W	10	6.9±4.7 (0−18.0)	10.1±5.5 (1.4−18.0)	10min
	Demons	D	10	6.4±4.4 (0−15.3)	9.3±5.3 (1.4−15.3)	45min
	Demons	W	20	6.8±4.6 (0−17.5)	10.4±5.5 (1.4−17.5)	2h 6min
	Demons	D	20	6.2±4.4 (0−15.3)	9.4±5.0 (1.4−15.3)	3h 2min
	Morphons	W	10	6.8±4.8 (0−16.9)	9.6±5.5 (1.4−16.9)	51min
	Morphons	D	10	6.4±4.3 (0−16.9)	9.1±5.8 (0−16.9)	1h 30min
	Morphons	W	20	7.0±4.7 (1.0−16.9)	10.1±5.4 (1.4−16.9)	2h 21min
	Morphons	D	20	6.3±4.3 (0−16.9)	9.1±5.7 (0−16.9)	4h 26min

### C. PET scan analysis

Analysis of residual uptake patterns measured on the FDG‐PET scans was performed for the patients with a good match after registration (Group 1 and 2); Group 3 was excluded from further analysis. To allow comparison with previous work,^(5)^ the contours corresponding to 34%, 40%, 50%, 60%, and 70% of ${\text{SUV}}_{\text{max}}$ high‐uptake values in the pre‐RT scans were outlined, based on the corresponding thresholds. The contours of 70%, 80%, and 90% of ${\text{SUV}}_{\text{max}}$ high‐uptake values were outlined, instead, on the post‐RT scans. Different thresholds were used for the pre‐ and post‐RT scans, because SUV values in tumor residue in the post‐RT PET scans are generally lower than SUV values in the tumor in the pre‐RT scan. The registration fields resulting from rigid and deformable registration of CT scans were applied to the contours in the nonregistered scans. Because the voxel values can change due to deformation and interpolation effects, the PET image was not deformed, but only the thresholded contours. Then, the DR between the contours in the (deformed) pretreatment and posttreatment thresholded PET volumes were determined according to the following definition:^(19)^

(1) $$DR=\frac{2\times V_{A\cap B}}{V_{A}+V_{B}}$$

where $V_{A}$ and $V_{B}$ represent the volumes of contours A and B, and $V_{A\cap B}$ represents their intersection. The DRs between volumes in post‐RT scans and rigidly registered pre‐RT scans were first evaluated. Then, also the DRs between volumes in the post‐RT and the deformed pre‐RT scans were calculated.

### D. Statistical analysis

Descriptive statistics of deformations were given in mean values ±1 standard deviation (SD). Paired t‐tests were performed to compare mean landmark distances before and after deformable registration. Wilcoxon signed‐rank tests were performed to compare the DR between the contours in the post‐RT PET scan and the pre‐RT (deformed) PET scan before and after deformable registration. P‐values smaller than 0.05 were assumed to be statistically significant.

## III. RESULTS

### A. Validation of registration fields

For the three patients with the 30 landmarks defined, the absolute distances between landmarks in the deformed pre‐ and post‐RT scans were calculated and summarized in Table 1 for rigid registration and for the eight deformable registration fields. Calculation times of the deformable registration fields ranged from 10 min to 4.5 hrs, depending on the algorithm and size of the VOI. An example of a single landmark selected in the pre‐ and post‐RT CT scans and tracked in the deformed pre‐RT scan for a patient in Group 1, is shown in Fig. 1. In the deformed pre‐RT CT scan the deformation field is indicated using arrows.

In five out of six cases, the mean landmark distances were smallest for the Morphons algorithm, with 20 iterations per scale and diffeomorphic field accumulation. But it also had the longest computation times, approximately 4.5 hrs per patient. Differences in mean landmark distances within the four variants of the Demons and Morphons algorithm were small, ranging between 0.4 mm and 0.9 mm, which is comparable to the in‐plane resolution of the CT scan. Although the landmark distances decreased between the best performing algorithm (either Demons or Morphons with 20 iterations per scale and diffeomorphic field accumulation) and the fastest Demons/Morphons algorithm (ten iterations per scale, weighted sum accumulation), this decrease was not statistically significant. Therefore, the fastest Demons variant and the fastest Morphons variant were selected for the subsequent analysis of all patients.

### B. Selection of best registration fields

For one of the 22 patients, deformable registration was not successful due to atelectasis that was not present in the pre‐RT scan and appeared on the post‐RT scan. In another patient, pneumonitis occurred at the location of the GTV in the post‐RT scan. These two patients were excluded from the analysis.

For the remaining 20 patients, the best deformable registration algorithm was determined based on the accuracy of the landmark analysis. Subsequently the patients were classified into 3 groups, as previously described. The mean absolute landmark distances for the patients in these groups are summarized in Table 2. For Group 1 and 3, the landmark distances decreased significantly after deformable registration (p<0.001 and p=0.025, respectively). The Morphons algorithm yielded the best results for six out of seven patients in Group 1, for three out of seven patients in Group 2, and for four out of six patients in Group 3.

**Table 2:** Mean absolute landmark distances for the three groups after rigid and after deformable registration. Mean ± SD of the mean absolute landmark distances of the patients in each group are shown after rigid, Demons, and Morphons registration. Also the numbers for the best deformable registration for each patient are shown. The p‐value results from comparing the absolute landmark distances after rigid registration to the distances after the best deformable registration

	*N*	*Rigid* (mean±SD)	*Demons* (mean±SD)	*Morphons* (mean±SD)	*Best Deformable Registration* (mean±SD)	*p‐value Rigid vs. Best*
Group 1	7	9.5±2.1 mm	5.4±1.7 mm	4.4±1.8 mm	3.8±1.2 mm	<0.001
Group 2	7	5.6±1.3 mm	4.9±1.1 mm	4.8±1.5 mm	4.5±1.1 mm	0.093
Group 3	6	13.6±3.2 mm	8.5±2.6 mm	10.2±2.7 mm	8.0±2.2 mm	0.025

N=number of patients per group.

### C. PET Dice overlap ratios

**Figure 2:**
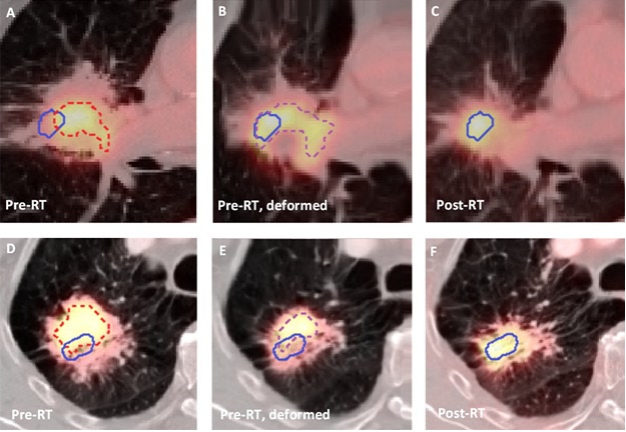
(Top) FDG‐PET/CT images of a patient in Group 1 with high overlap fractions: pre‐RT scan (a), deformed pre‐RT scan (b), post‐RT scan (c). The 60% of ${\text{SUV}}_{\text{max}}$ contour in the pre‐RT scan (red, dashed), the deformed 60% of ${\text{SUV}}_{\text{max}}$ contour in the deformed pre‐RT scan (purple, dashed), and the 70% of ${\text{SUV}}_{\text{max}}$ in the post‐RT scan (blue) are depicted. (Bottom) FDG‐PET/CT images of a patient in Group 1 with low overlap fractions: pre‐RT scan (d), deformed pre‐RT scan (e), rigidly registered post‐RT scan (f). The 60% of ${\text{SUV}}_{\text{max}}$ contour in the pre‐RT scan (red, dashed), the deformed 60% of ${\text{SUV}}_{\text{max}}$ contour in the deformed pre‐RT scan (purple, dashed), and the 70% of ${\text{SUV}}_{\text{max}}$ in the post‐RT scan (blue) are depicted.

For six of the seven patients in Group 1, large overlap between pre‐ and post‐RT high FDG‐uptake areas occurred. In Fig. 2, the (deformed) PET/CT scans of two patients in Group 1 are shown: the (deformed) 60% of ${\text{SUV}}_{\text{max}}$ contours in the (deformed) pre‐RT scan are compared to the 70% of ${\text{SUV}}_{\text{max}}$ contour in the post‐RT scans. For the first patient (Figs. 2(a) and (c)) the tumor shape in the deformed scan visually better resembles the shape after RT. Also, in this slice, the 70% of ${\text{SUV}}_{\text{max}}$ contour in the post‐RT scan is almost entirely located within the deformed 60% of ${\text{SUV}}_{\text{max}}$ contour, as opposite to the nondeformed pre‐RT contour. After deformation of the pre‐RT contour, the DR between the described contours for this patient increased from 6% to 21%. For the second patient (Figs. 2(d) and (f)), visual evaluation of the scans shows that the tumor's shape in the deformed pre‐RT scan is quite similar to the post‐RT scan. However, the 70% of ${\text{SUV}}_{\text{max}}$ contour in the post‐RT scan appears to have low overlap with the 60% of ${\text{SUV}}_{\text{max}}$ contour in the deformed pre‐RT scan. Comparing the scans, it seems that the hotspot in the pre‐RT scan did not move to a new location, but a new hotspot has shown up in the post‐RT PET‐scan. The DR for this patient decreased from 19% to 12% after deformation of the pre‐RT contour.

**Figure 3:**
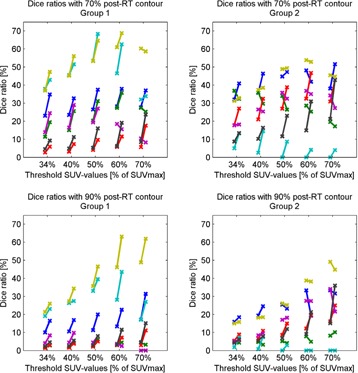
Dice overlaps ratio of FDG uptake areas. For each patient (represented by different colors) paired values are plotted, before (left cross in the pair) and after (right cross) deformable registration for Group 1 and 2, connected by a line. Left, top to bottom: DRs of the (deformed) pre‐RT contours with the 70% and 90% of ${\text{SUV}}_{\text{max}}$ contours in the post‐RT scan for Group 1. Right, top to bottom: DRs of the (deformed) pre‐RT contours with the 70% and 90% of ${\text{SUV}}_{\text{max}}$ contours in the post‐RT scan for Group 2.

In Fig. 3, the DRs for the 70% and 90% of ${\text{SUV}}_{\text{max}}$ contours in the post‐RT scan are shown; DRs for the 80% of ${\text{SUV}}_{\text{max}}$ threshold are similar (not shown). For Group 1, deformable registration of the pre‐RT PET scan significantly increased almost all DRs compared to rigid registration; no significant difference was found for the 70% of ${\text{SUV}}_{\text{max}}$ pre‐RT contour and the 70%, 80%, and 90% of ${\text{SUV}}_{\text{max}}$ post‐RT contour, and for the 60% of ${\text{SUV}}_{\text{max}}$ pre‐RT contour and the 90% of ${\text{SUV}}_{\text{max}}$ post‐RT contour. The largest improvements were achieved for the 70% of ${\text{SUV}}_{\text{max}}$ in the post‐RT scan for this group. For Group 2, significant differences were only found for the DRs of the 34% of ${\text{SUV}}_{\text{max}}$ pre‐RT contours, and the 80% and 90% of ${\text{SUV}}_{\text{max}}$ post‐RT contour.

## IV. DISCUSSION

Deformable registration fields need to be validated and the accuracy needs to be estimated before implementation in research or clinical practice.^20^, ^21^ This work is, to our knowledge, the first study investigating the accuracy of image registration between pre‐RT and follow‐up imaging using scans that are four months apart. There are multiple deformation algorithms available nowadays that are utilized for several purposes: tumor tracking during the respiratory cycle,^(8)^ contour propagation for adaptive RT,^(22)^ and dose accumulation.^(10)^ Validation datasets have been annotated with landmarks mainly for 4D CT imaging of the thorax,^(23)^ but also other treatment sites and organs, are becoming available,^(12)^ but none are described for follow‐up imaging. De Moor et al.^(24)^ used a rigidity constraint to steer the deformation field calculation for PET to PET registrations in such a way that the tumor volume of interest remains constant for the purpose of response assessment. For CT to CT registration, this might also be an option to steer the deformation field calculation in regions with lower CT contrast; this needs, however, further investigation regarding the accuracy of such an approach. Another option is the use of synthetic datasets for validation purposes.^(20)^ Once deformation algorithms are validated, they could also have potentially a role in dose assessment in a reirradiation setting,^(25)^ for example in lung cancer, where large anatomical variations occur after treatment. The actual dose delivered to every organ can then serve as an indication of the dose level, and the actual high‐dose region inside that organ that might then guide the treatment plan optimization for reirradiation.

In the first part of this work, we have chosen a landmark‐based verification to extensively study three patients comparing eight deformable registration fields, based on Demons and Morphons, in order to estimate the accuracy of the various algorithms. Using the best performing algorithms, a significant improvement, compared to rigid registration alone, was achieved for 70% (14 out of 20) of the patients. It should be noted that for the remaining 30% of the patients, deformable registration did not significantly improve. This happened mainly for extremely large anatomy variations both for normal lung and tumor. In this case, modification of the deformation algorithms to take missing tissue into account might be necessary.^(26)^ Also, for about two‐thirds of the patients, the Morphons algorithm showed smallest deviations, while for one‐third of them, it was Demons. Since we could not identify any causes why for some patients Morphons and for others Demons was the best, it is still necessary to perform individualized validation in order to determine which algorithm is the most accurate for each individual patient.

Although specific nonrigid registration algorithms exist that incorporate tissue properties (Al‐Mayah et al.^(27)^), the frequently used (mathematical) nonrigid registration algorithms do not model physical properties of organs or tumors. The algorithm will search for matching intensity values inside images, but could also try to match parts of the tumor that were in regression and started growing again. The deformation vector from the original tumor voxel to a newly grown tissue might look correct on an imaging level, but will represent different biological tissues. More frequent follow‐up imaging and characterization of tumor regression patterns are needed to tackle these issues.

To perform response assessment of treatment on a local or voxel level, multiple strategies can be used. One of these techniques models the voxel control probability and could benefit from better image registration techniques.^28^, ^29^ However, only few of the described methods have validated their registration accuracy prior to building their predictive models. In our study, the DR between pre‐ and post‐RT FDG‐uptake increased after deformable registration. As expected for Group 2, the deformed pre‐RT CT scan showed no significant reduction in average landmark distance compared to rigid registration and, hence, the DR showed no improvement. This confirms our hypothesis that a successful deformable registration increases the overlap ratio between high FDG‐uptake areas in pre‐ and post‐RT PET scans. This further implies that FDG‐uptake is a suitable target for dose‐boosting/painting as an indicator of treatment‐resistant regions inside the tumor.^(30)^ Since image deformation and subsequent interpolation of PET scans may change the actual SUV values, it was decided to apply the deformation fields to the contours instead of the image datasets. In this study, PET contours were delineated using thresholding, but other techniques, like gradient‐based segmentation,^(31)^ are available. This technique may yield more robust segmentation of the PET values and could be used in a future research.

The proposed technique is also directly applicable to other tracers. Bowen et al.^(32)^ showed a tri‐modality investigation linking proliferation and hypoxia PET to residual FDG‐uptake. Although they limited the registration process to an affine registration, it shows the possibility of finding imaging and dose response relationships between multiple modalities. Hypoxia is another factor contributing to treatment resistance^(33)^ and does not necessarily coincide with metabolic active areas. Therefore, the tumor might recur or have a residue in the hypoxic area, which cannot be predicted from the FDG‐PET scan. Combining hypoxia and FDG‐PET imaging could give more insight in the areas of treatment resistance.^(28)^ The presented methodology could then directly be applied to pre‐RT hypoxia PET imaging and the assessment of treatment recurrence.

## V. CONCLUSIONS

We implemented a clinical validation framework for individualized voxel‐to‐voxel deformable registrations between pre‐ and post‐RT PET/CT scan of lung cancer patients. Deformable registration significantly improved registration quality between pre‐ and post‐RT CT scans, but the quality of the registration needs to be evaluated for all patients individually. DR of pre‐ and post‐RT high FDG‐uptake regions increased using deformable registration, depending on the registration quality. This technique allows more precise assessment in follow‐up imaging of the initial location where tumor relapses occur.

## ACKNOWLEDGMENTS

This study was performed within the framework of CTMM, the Center for Translational Molecular Medicine (http://www.ctmm.nl), Project AIRFORCE No. 03O‐103. We would like to thank Georgy Shakirin for initial discussions on the study design. One of the authors (W.v.E.) would like to acknowledge funding (KWF MAC 2011‐4970) from the Dutch Cancer Society.

## Supporting information

Supplementary Material
