# Supplementary material for: Validation of nonrigid registration in pretreatment and follow‐up PET/CT scans for quantification of tumor residue in lung cancer patients
Source: J Appl Clin Med Phys. 2014 Jul 8;15(4):240–50. doi: 10.1120/jacmp.v15i4.4847 (PMC5875523; doi:10.1120/jacmp.v15i4.4847)
Supplement: Supplementary file 1 — Supplementary Material [file ACM2-15-240-s001.doc]

**Title: Validation of non-rigid registration in pre-treatment and follow-up PET/CT scans for quantification of tumor residue in lung cancer patients**

**Authors:** **Jolanda Spijkerman^1^, Davide Fontanarosa^1, 2^, Marco Das^3^, Wouter van Elmpt^1^**

^1^Department of Radiation Oncology (MAASTRO), GROW – School for Oncology and Developmental Biology, Maastricht University Medical Centre, Maastricht, The Netherlands

^2^Philips Research, X-ray Imaging Systems, Eindhoven, The Netherlands

^3^Department of Radiology, GROW – School for Oncology and Developmental Biology, Maastricht University Medical Centre, Maastricht, The Netherlands

Author for correspondence: Davide Fontanarosa

Maastro Clinic, Dr Tanslaan 12, 6229ET Maastricht, the Netherlands

cell: +31 (0) 6 11534106

email: davide.fontanarosa@maastro.nl

**Running Title:** Validation of non-rigid registration for tumor residue quantification
